# Pain Management in the Elderly: Transdermal Fentanyl for the Treatment of Pain Caused by Osteoarthritis of the Knee and Hip

**DOI:** 10.1155/2014/262961

**Published:** 2014-01-05

**Authors:** Sylwester Mordarski

**Affiliations:** Department of Anaesthesiology and Intensive Care, Pain Clinic, University of Medicine, Chalubinskiego 1a, 50-368 Wroclaw, Poland

## Abstract

This study was designed to evaluate the utility of transdermal fentanyl (transdermal fentanyl, TDF) for the treatment of pain due to osteoarthritis (osteoarthritis, OA) of the knee and hip, which was not adequately controlled by nonopioid analgesics or weak opioids. WOMAC is a reliable, valid, and responsive multidimensional, self-administrated outcome measure designed specifically to evaluate patients with OA of the knee or hip. TDF significantly increased pain control and improved functioning and quality of life. Metoclopramide appeared to be of limited value in preventing nausea and vomiting.

## 1. Introduction 

Osteoarthritis (OA) is a slowly progressing disease, characterized mainly by degeneration of articular cartilage which is manifested clinically by gradual increase of pain, stiffness, and reduction of mobility. OA affects also articular capsule being the main reason of musculofascial pain and limitation of everyday activities. About 40–60% of patients with radiologically confirmed lesions suffer from pain, stiffness, and reduction of mobility in the affected joint, and about 55% of patients say that pain is the worst aspect of the disease [1]. OA is inseparably linked with general increase of elderly population, slowly gaining socioeconomic significance. It is estimated that, in Poland, about 4 millions of people suffer from it, and aging of the society and other factors (e.g., overweight) will still increase this number.

Diagnosis of osteoarthritis is based, most of all, upon clinical evaluation and a radiological picture. Radiological lesions occur most often in the sites of the highest load. In the advanced osteoarthritis, deformity of articular ends of bones occurs. They have forms of fungus-like deformities, oblateness of epiphyses. There is abnormal, varus, or valgus position of epiphyses, subluxation, or dislocation of the joint [2, 3]. The especially important radiological symptom is narrowing of articular space as a result of gradual loss of articular cartilage [4]. Apart from other characteristic radiological symptoms (subchondral sclerosis, cysts, and changes of joint outline), the results of laboratory examinations are considered, which may support the diagnosis of this disease (among other things, analysis of articular fluid).

Before occurrence of radiological lesions, the diagnosis of osteoarthritis is difficult. Therefore the American College of Rheumatology [5] proposed criteria which, with use of appropriate algorithms, allow approximation of diagnosis. The evaluation of a degree of clinical progression does not always go hand in hand with a radiological picture. It is not strange that in almost every person above 40 years of age, radiological lesions are found, but clinical symptoms occur only in every third person.

In OA treatment, the stopping of deterioration of disease is of prime importance, facilitation of patient's gait and articular mobility by a decrease of pain, what influences quality of life. In the individual treatment plan, pharmacological and nonpharmacologic treatment are taken into consideration, for example, physiotherapy, mechanical aids, and surgical treatment. In current recommendations of American College of Rheumatology [5–7] concerning treatment of osteoarthritis, first of all, paracetamol is suggested, in monotherapy or in combination with, for example, codeine/tramadol in order to relieve pain and in coexisting inflammation, nonsteroidal anti-inflammatory drugs (NSAIDs) acting peripherally analgetically and anti-inflammatory. They are recommended for general and local use [8, 9]. Depending on a disease course, treatment may be extended by steroidal drugs (most commonly used locally), hialuronic acid, and local anestethics.

*Purpose of the Study.* Clinical question is, what is efficacy of transdermal fentanyl in pain relief and clinical symptoms relief in patients with osteoarthritis, most of whom used paracetamol/NSAIDs before?

## 2. Clinical Material and Method 

*Patient Selection.* The following persons were included in the study:

- ambulatory treated persons aged ≥55 years with osteoarthritis of the knee or hip in 2° and 3° of radiological lesions according to Altman,
- pain intensity during walking expressed by WOMAC index ≥80 mm (per 100 mm of the scale),
- lack of satisfactory pain control after inclusion of tramadol within 1 week,
- deterioration of disease symptoms in the opinion of the physician by ≥1 point in 5-point Likert scale,
- persons qualified for surgery.

Exclusion criteria are

- patients after arthroscopic procedures within last 3 months,
- patients receiving intra-articular steroids within last 3 months,
- diseases increasing the risk of complications related to administration of opioids,
- opioid drugs intolerance.

Patients with the liver, kidney, cardiac, and endocrine glands diseases, haematologic diseases, asthma, and/or chronic obstructive bronchial disease were excluded from the study. During the study, administration of antirheumatic drugs and analgesics was prohibited as well as muscle relaxant drugs excluding paracetamol used as needed.

Comparison of demographic data and data obtained from history taking at the beginning of treatment was shown in Table 1.

**Figure 1:**
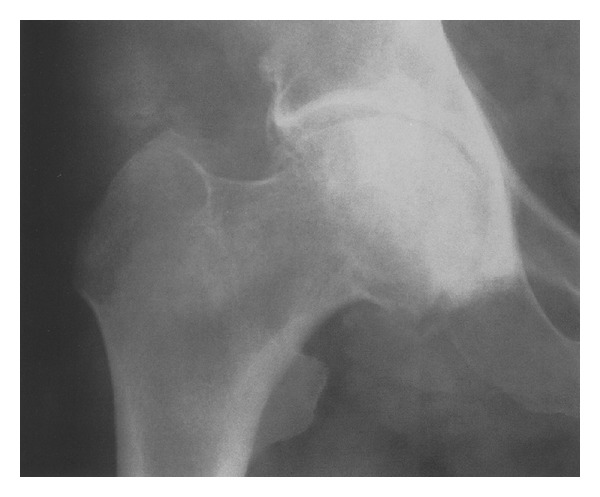
Osteoarthritis of the hip (3° of Altman scale, anteroposterior fluoroscopic view).

**Figure 2:**
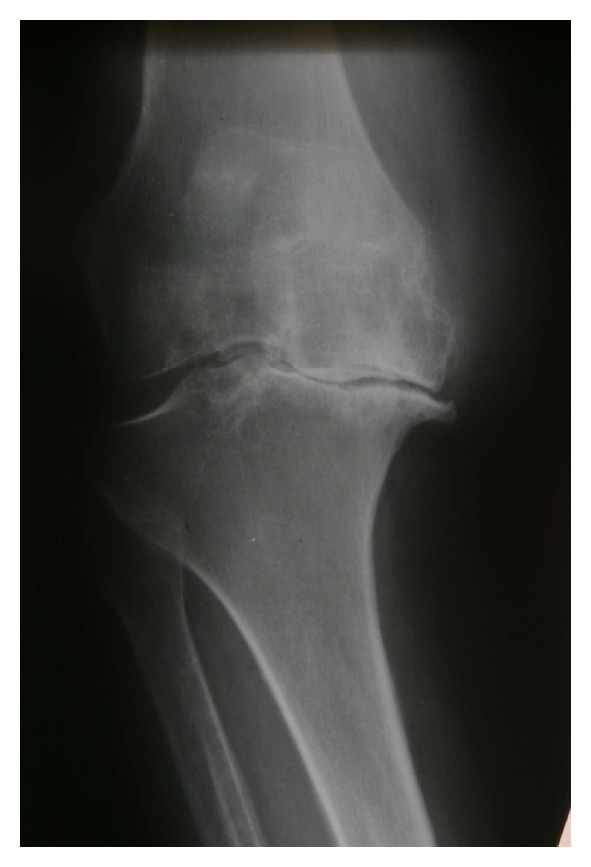
Osteoarthritis of the knee (3° of Altman scale, anteroposterior fluoroscopic view).

Figures 1 and 2 show examples of radiological lesions of the treated patients.

*Description of the Intervention*. After inclusion into the study, the patients in the first week of treatment received paracetamol + tramadol in generally recommended doses. Insufficient pain and clinical symptoms control qualified for stopping current treatment and inclusion of transdermal fentanyl in the following week with a release of 25 mcg/hr of the active substance. The patches were replaced every 72 hours. Participants were encouraged to take metoclopramide (supplied as 10 mg tablets) immediately if they experienced any nausea or vomiting. They were also encouraged to take a laxative if they had constipation.

*Evaluated Parameters*. Principal, pain, stiffness, and activity of the affected joint are expressed by WOMAC index (Western Ontario and McMaster Universities Osteoarthritis Index—WOMAC index) [10]. This index refers to subjective patient's feeling. In part A of WOMAC index, the patient defines intensity of his/her own pain on a visual analogue scale (visual analogue scale, VAS, where 0 signifies “no pain” and 100 “worst pain imaginable”): at night, during walking and at rest, morning stiffness of the joint and total evaluation of pain intensity (5 questions); in part B, stiffness of the joint (2 questions) and in part C, physical ability (17 questions).

Additionally, treatment efficacy was evaluated by a treating person on 5-point Likert scale [11].

All the above mentioned parameters were evaluated in the beginning (in the frame of the initial examination), in one week after treatment start, four weeks after treatment start, and after the end of treatment (at least six weeks, and 10 weeks the latest after the start of treatment). This observation period, as observations show, is sufficient for proper evaluation of treatment efficacy.

In the beginning of observation and during pharmacotherapy, the physician evaluated the degree of reduction of joint function based on main disease symptoms (pain in the beginning of movement, pain at movement/joint load, fatigue pain, and feeling of tension/stiffness), noted down the first improvement of joint function, and evaluated treatment result after its end (in a scale: very good, good, moderate, no change, and deterioration). The end of treatment was a procedure of plastic surgery with endoprosthesis of the damaged joint in every patient. bioethics commission approved the study.

## 3. Results 

During 10-week treatment, a significant decrease in value of global WOMAC index was observed as well as sub scale values, that is, group of questions about pain and stiffness in relation to starting values.

Functional index WOMAC, which describes physical function of the impaired joint based on 17 details with consideration of difficulties in everyday life of the patient, had similar values in both groups in the beginning of treatment.

**Figure 3:**
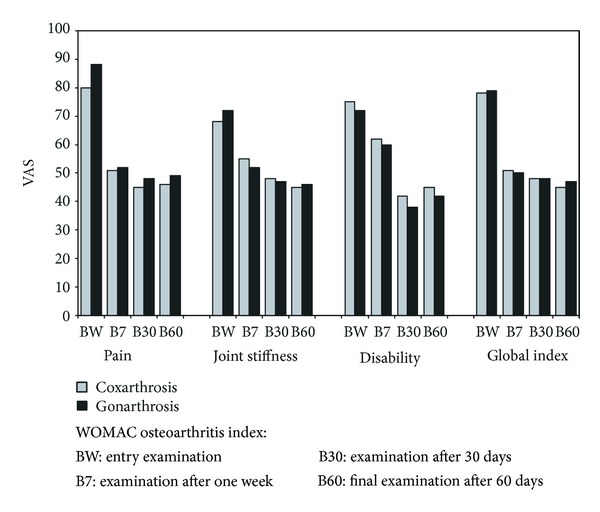
General improvement in WOMAC index (global index) and improvement of pain, stiffness, and function before and on different times after therapy start.

Exactly, these situations in which patient's ailments were especially severe may be considered the leading symptoms. Thus, the highest initial pain scores (in the limits of 60–80 mm in VAS scale) were found in categories of such activities as “descending/climbing the stairs”, “bending down to the floor”, and “tiring home works”. At the same time, exactly in these categories, the improvement of indexes was the highest (between 20 and 27 mm) (Figure 3).

**Figure 4:**
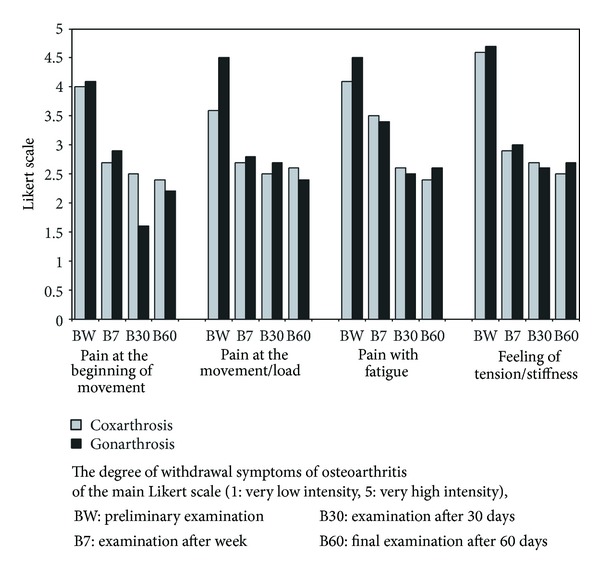
A degree of withdrawal of osteoarthritis symptoms of Likert scale.

Other limitations of function were reported by the patients regarding situation in which locomotor efficiency and stable balance are required, as for example, with “getting on/getting off the car”, “getting into and getting off the bathtub”, or “getting off the bed or chair”. In general, it should be indicated that in all 17 categories, after 10 weeks of treatment, a significant improvement of function of impaired joint occurred, while after 6 weeks, the obtained effects in both groups were similar. The confirmation of the improvement of impaired joint mobility and a decrease of pain upon movement were shown also in a study with the use of the Likert scale (improvement in the range of 1.6 and 2.0 points) (Figure 4).

Now, it should be concluded that similar improvement was observed by the team carrying out rehabilitation activities (not included in the study).

The observed adverse events of fentanyl use were shown in Table 2.

Nausea and somnolence were of prime importance among adverse events. Metoclopramide was effective for nausea or vomiting. It was included on the first day of treatment, predicting occurrence of these symptoms, at a dose of 20–30 mg/daily p.o. Excessive somnolence did not requir treatment and subsided spontaneously in 7–10 days after treatment started. Each patient was carefully examined before inclusion into the study and informed in details about possible adverse events resulting from use of transdermal fentanyl.

The statistical analysis of the obtained results was carried out with the use of Statistica 5.0 PL software. A nonparametric test for dependent variables was used, the Wilcoxon test of matched pairs. The selection of the test resulted from the character of analyzed variables. The differences were accepted as statistically significant at the level of significance of *P* < 0.05.

In both examined groups of patients, significant differences were determined at the level of WOMAC index regarding pain after 7 days, after 30, and after 60 days compared to the initial level, regarding stiffness, after 7 days, after 30, and after 60 days compared to initial level, regarding function, after 30 and after 60 days compared to baseline and regarding global index, after 7 days, after 30 days, and after 60 days compared to baseline. Statistically significant differences regarding the level of WOMAC index between observations were not determined.

In the analysis of a degree of subsiding of main osteoarthritis symptoms determined in 5-point Likert scale, statistically significant differences were determined in both groups compared to initial observations, regarding pain intensity in the beginning of movement in examination after 7, after 30, and after 60 days, regarding pain intensity at movement/load in the examination after 7, after 30, and after 60 days, regarding pain intensity with fatigue after 30 and after 60 days, and regarding the level of tension/stiffness in the examination after 7, after 30, and after 60 days. Only in pain intensity in the beginning of movement, in a group of patients with osteoarthritis of the knee, onset of statistically significant decrease compared to the level of this parameter in study after 7 and 60 days was determined.

## 4. Discussion 

Due to lack of causal treatment of osteoarthritis of the knee or hip, pharmacologic treatment is, on one hand, directed to pain relief, on the other hand—due to treatment of inflammation—to the inhibition of progression of impairment. The used pharmacotherapy includes analgesics and anti-inflammatory drugs, NSAIDs. Clinical trials carried out in order to show the efficacy of any method of therapy use usually evaluation criteria of ailments experienced by examined patients. Often, in OA treatment, NSAIDs are used, included by many centres in the standard procedure. Regarding the fact that the purpose of use of these drugs is a decrease of pain and maintenance of locomotor ability and minimization of functional reduction, at the same time, these drugs may cause adverse events (especially concerning alimentary tract) which may also lead to a decrease of quality of life; the individual, subjective evaluation of patient well-being plays a significant role [7, 12–16]. Severe, suddenly occurring pain may cause avoidance of physical activity by the patient and moving away from different aspects of social activity. Thus, the purpose of treatment should be, among other things, a qualitative and quantitative decrease of pain, so that locomotor activity is maintained in everyday life. With the use of multidimensional WOMAC questionnaire, a significant decrease of typical articular ailments after the use of transdermal fentanyl due to which final treatment predicted in the future surgery enables patients to take part in locomotor rehabilitation activities [17, 18].

WOMAC questionnaire is an evaluation instrument specific for osteoarthritis [10]. It is characterised by a good correlation between the results of the questionnaire, a degree of progression of radiological lesions, a deficit in extension, and limitation of flexion of the joint. It is known from earlier studies on WOMAC scale that there is a clear relationship between reduction of activity of the joint and psychosocial variables such as limitation of everyday home and professional activities and social integration in the family and circle of friends [19].

Start of treatment with potent opioids requires the detailed information for the patient about possible and expected drug adverse events from treating person. Patients receiving strong opioids might require rapid dosage escalation to achieve satisfactory pain relief. Dosage escalations might reduce the benefit/risk ratio of opioid treatment and the potential for improvements in functional capacity and quality of life that opioid treatment can provide. The transdermal opioid formulations have been developed to guarantee long-term pain relief and avoid excessively high serum opioid concentrations. It clearly decreases patient anxiety and increases safety of the therapy [20].

The above report should be considered as initial. It regards a small group of patients treated in a limited period of time (ultimately—treatment end—arthroplasty of the damaged joint). Randomized multicentre study should be designed in order to prove efficacy and safety of the proposed treatment.

## 5. Conclusions

1. Treatment of pain in advanced stages of osteoarthritis with the use of transdermal fentanyl leads to clear improvement of mobility of increasing of efficiency of joint function, which is related to improvement of quality of life of the patient.
2. The observed adverse events occur mainly in the first week of treatment. Nausea, vomiting decreases with simultaneous use of metoclopramide, and the other—somnolence, dizziness, and so on subsided gradually upon treatment.

## Figures and Tables

**Table 1:** Osteoarthrosis-demographic data and data obtained from history taking in the beginning of treatment.

Criterion	Hip joint	Knee joint
Number of patients	9	39
Sex		
Female	6	25
Male	3	14
Age (years—mean)	63.4	60.7
Clinical symptoms		
Arthritis	100%	100%
Reduction of mobility	88.8%	98.5%
Crepitations in joints	44.4%	62.0%
Radiological symptoms		
Narrowing of articular fissure	88.8%	68.0%
Irregularities of articular surface	33.3%	37.4%
Sclerosis/osteophyte	44.4%	30.5%
Main causes of osteoarthrosis		
Loading/overloading	55.5%	67.3%
Injuries	22.2%	21.7%
Defect of cartilage	11.1%	20.4%
Metabolic disturbances	22.2%	14.2%
Duration of disease (years)		
<2	—	33.5%
2–5	—	31.6%
5–10	55.5%	21.9%
>10	44.4%	13.0%
Engaged joint		
Right	66.6%	49.1%
Left	33.3%	29.2%
Both	—	21.2%

**Table 2:** The observed adverse events.

The observed adverse events % (*n* = 48)	
Nausea	32%
Vomiting	26%
Somnolence	16%
Dizziness	9%
Weakness	6%
Itching of skin	5%
